# Antipsychotic-Induced Remodeling of the Gut Microbiota: Drug-Specific Patterns and Molecular Links to Metabolic Adverse Effects

**DOI:** 10.3390/ijms27198471

**Published:** 2026-09-23

**Authors:** Răzvan-Ioan Papacocea, Miculescu Adina Gabriela, Volosin Radu-Ștefan, Thais Russomano, Ioana Raluca Papacocea

**Affiliations:** 1 Department of Psychiatry II, Faculty of Medicine, Carol Davila University of Medicine and Pharmacy, 020021 Bucharest, Romania; 2 “Dr. Carol Davila” Central Military Emergency University Hospital, 010825 Bucharest, Romania; 3 Center for Innovation and eHealth, Carol Davila University of Medicine and Pharmacy, 030167 Bucharest, Romania; adina-gabriela.miculescu0721@stud.umfcd.ro (M.A.G.); radu-stefan.volosin2024@stud.umfcd.ro (V.R.-Ș.); raluca.papacocea@umfcd.ro (I.R.P.); 4 Center for Aerospace Medicine Studies (CEMA), University of Lisbon, 1649-004 Lisbon, Portugal; thais@innovaspace.org; 5 Space Technology Center, AGH University of Kraków, 30-054 Kraków, Poland; 6 Discipline of Medical Physiology, Faculty of Medicine, Carol Davila University of Medicine and Pharmacy, 020021 Bucharest, Romania

**Keywords:** antipsychotics, gut microbiota, schizophrenia, metabolic syndrome, gut–brain axis, pharmacomicrobiomics

## Abstract

Antipsychotic drugs are essential for the treatment of schizophrenia-spectrum disorders and other severe psychiatric conditions, but several agents produce clinically important metabolic and gastrointestinal adverse effects. Increasing evidence suggests that these outcomes may involve bidirectional interactions between antipsychotics and the gut microbiota. This structured narrative review integrates human, animal, gnotobiotic, in vitro, pharmacokinetic, and multi-omic evidence to examine drug-specific microbiome effects and microbiota-dependent modulation of host responses. Olanzapine has the strongest mechanistic evidence, with germ-free, antibiotic-depletion, colonization, and vagotomy studies supporting a contributory role of the microbiota in weight gain, adiposity, inflammation, altered microbial metabolites, and lipid dysregulation. Risperidone also shows microbiota-dependent metabolic and neurobiological effects, whereas findings for clozapine, quetiapine, aripiprazole, lurasidone, and amisulpride remain more heterogeneous or limited. Across agents, no universal taxonomic pattern has emerged. Functional pathways involving short-chain fatty acids, bile acids, epithelial barrier integrity, immune and neuroendocrine signaling, vagal pathways, and microbial drug metabolism appear more informative than individual taxa or the Firmicutes/Bacteroidota ratio. Current evidence remains limited by methodological heterogeneity, small cohorts, confounding, and predominantly preclinical mechanistic data. Prospective, treatment-naive, drug-specific longitudinal multi-omic studies are required.

## 1. Introduction

Antipsychotic drugs remain the first line of pharmacological treatment for schizophrenia-spectrum disorders (SSDs) and are also used across several other psychiatric conditions, including bipolar disorder and major depressive disorder. Despite their substantial clinical efficacy, their use is frequently limited by adverse effects, including weight gain, insulin resistance, dyslipidemia, altered appetite, gastrointestinal hypomotility, constipation, and, for some compounds, a considerable increase in cardiometabolic risk [1,2]. The magnitude and timing of these adverse effects vary across drugs and individuals. Although mechanisms involving central and peripheral dopamine, serotonin, histamine, muscarinic, and adrenergic receptors account for part of this liability, receptor-based models alone do not fully explain the pronounced variability and the distinct metabolic profiles of individual antipsychotic compounds [1,3]. The gut microbiota has emerged as a potential contributor to this heterogeneity. It participates in the regulation of energy harvest, epithelial barrier integrity, bile-acid metabolism, immune maturation, endocrine signaling, and the generation of neuroactive and metabolically active molecules [3,4]. Microbial metabolites and structural components, including short-chain fatty acids (SCFAs), tryptophan-derived metabolites, secondary bile acids, branched-chain fatty acids, and lipopolysaccharide (LPS), can influence host metabolic, immune, neuroendocrine, and neural signaling pathways, including glutamatergic, GABAergic, serotonergic, and dopaminergic systems [3,5]. Antipsychotics may perturb this ecosystem through several potentially overlapping mechanisms. Some compounds exert direct antimicrobial or growth-inhibitory effects on commensal microorganisms [6]. Moreover, indirect effects may arise through drug-induced changes in appetite and dietary intake, gastrointestinal motility and transit, host metabolism, epithelial barrier function, and mucosal immune signaling [4,7]. Conversely, the gut microbiota may affect xenobiotic disposition through direct microbial biotransformation and modulation of host drug-metabolizing pathways, potentially altering systemic drug exposure, therapeutic response, and tolerability [8,9]. Nevertheless, direct evidence for microbiome-mediated effects on antipsychotic pharmacokinetics in humans remains limited. Interpretation of antipsychotic-associated microbiome alterations is further complicated by evidence that similar changes are also observed in antipsychotic-naive or medication-free patients with schizophrenia, suggesting that factors beyond medication exposure may contribute to these microbial alterations [10,11,12,13]. Studies in untreated cohorts have reported differences in microbial community structure and functional pathways involving SCFA metabolism, glutamate-GABA signaling, tryptophan-kynurenine metabolism, immune regulation, and lipid metabolism, although specific taxonomic patterns have not been fully consistent across populations [10,11,12,13]. Fecal microbiota transfer from patients with schizophrenia to recipient rodents has also produced behavioral and neurochemical alterations, including changes in glutamate-glutamine-GABA cycling and tryptophan-kynurenine metabolism [14,15]. These experiments support the biological plausibility of a microbiome contribution to disease-associated phenotypes but do not establish causality in humans. Consequently, microbiome profiles observed in medicated patients may reflect varying contributions from the underlying disorder, previous and current drug exposure, metabolic phenotype, diet, gastrointestinal physiology, smoking and other lifestyle factors, sex, geography, and interactions among these determinants [3,10,12]. For clarity, microbiome changes found in antipsychotic-naive or medication-free patients are considered mainly disease-associated, whereas changes appearing after treatment initiation are considered treatment-associated. In chronically treated patients, however, these two influences are difficult to separate.

The present review examines the bidirectional relationship between antipsychotic exposure and the gut microbiome, with a focus on drug-specific microbial changes and their potential links to metabolic adverse effects. We distinguish, where possible, treatment-emergent alterations from disease-associated microbiome features and compare evidence across individual antipsychotic compounds. Particular attention is given to the strength and consistency of findings from human longitudinal studies, cross-sectional observations, animal models, gnotobiotic experiments, and in vitro studies. This approach allows us to highlight reproducible patterns, major sources of heterogeneity, and key gaps that currently limit the clinical application of microbiome-informed strategies in antipsychotic treatment. A conceptual overview of the bidirectional interactions between antipsychotic exposure, gut microbiota remodeling, host pathways, and clinical outcomes is shown in Figure 1.

## 2. Antipsychotic–Microbiota Interactions: Evidence and Mechanisms

The included studies reported variable changes in gut microbial diversity, composition, and function following antipsychotic treatment. Table 1 summarizes the main bacterial changes associated with each antipsychotic drug, together with the study design and the principal microbiota-related findings.

### 2.1. Overall Patterns of Antipsychotic-Associated Microbiome Changes

Across the evidence base, antipsychotic exposure appears to affect microbial community composition and functional outputs more consistently than alpha diversity itself [8,10]. Alpha-diversity responses remain heterogeneous. One longitudinal metagenomic study in bipolar depression reported increased microbial diversity after one month of quetiapine treatment [41], whereas a prospective study in patients with depression or anxiety found that antipsychotic exposure, and particularly higher antipsychotic doses, was associated with lower Shannon and phylogenetic diversity [53]. Cross-sectional studies have also reported lower microbial diversity among women receiving atypical antipsychotics, an association not consistently observed in men [36,49]. Taxonomic changes are similarly heterogeneous. Reductions in *Bacteroidota*, enrichment of Proteobacteria, alterations in butyrate-producing taxa, and shifts in the Firmicutes/Bacteroidota ratio have each been reported, but none constitutes a reproducible pattern across drugs, populations, and experimental models [10,16,54]. The Firmicutes/Bacteroidota ratio should therefore be regarded as a context-dependent ecological descriptor rather than a drug-specific mechanistic biomarker. It should not be interpreted as a universal clinical biomarker or used in isolation to infer treatment-related metabolic risk. Collectively, the available evidence supports 3 non-exclusive pathways: direct drug–microbe interactions, indirect remodeling of the intestinal environment through host pharmacology, and secondary microbial changes accompanying drug-induced metabolic dysfunction [7,8]. Their relative contribution likely depends on the individual antipsychotic, baseline microbial ecosystem, sex, diet, dose, treatment duration, and anatomical site sampled.

### 2.2. Molecular Mechanisms Linking Antipsychotics, Microbiota, and Host Phenotypes

Evidence for direct antimicrobial activity is compound- and assay-specific rather than a uniform class property. Aripiprazole inhibits the growth of several representative intestinal commensals in vitro, although inhibitory concentrations may exceed those achieved systemically during clinical treatment [45]. Olanzapine has also shown growth-inhibitory activity against *Escherichia coli* and *Enterococcus faecalis* in vitro, but these experiments used supraphysiological concentrations, limiting direct extrapolation to the human intestinal environment [17]. More broadly, a large-scale screen of human-targeted non-antibiotic drugs demonstrated that antipsychotic compounds were prominently represented among drugs capable of inhibiting human gut bacterial isolates [50]. Translation of such findings to in vivo conditions remains uncertain because intestinal drug exposure is influenced by oral absorption, solubility, food and mucus interactions, intestinal transit, biliary excretion, enterohepatic cycling, and microbial metabolism. Consequently, microbiome remodeling during treatment may result from both direct luminal drug exposure and indirect host-mediated effects [7,9]. Changes in microbial metabolic output, particularly SCFAs, are among the most frequently implicated functional consequences of antipsychotic-associated microbiome remodeling. Experimental olanzapine studies have demonstrated concurrent changes in microbial composition, SCFA-related pathways, body weight, lipid metabolism, and inflammatory phenotypes [18], while clinical multi-omic analyses have identified altered microbial and fecal metabolic profiles in association with antipsychotic-induced weight gain [16]. Nevertheless, the direction of individual SCFA changes varies between models, and the abundance of putative SCFA-producing organisms should not be considered equivalent to directly measured metabolite production. Experimental manipulation of the microbiota provides stronger evidence for a functional contribution to host metabolic effects. Antibiotic depletion attenuated olanzapine-associated weight gain, adipose accumulation, circulating free fatty acids, adipose inflammatory markers, and hepatic lipogenic signaling in rats [19]. In a complementary risperidone model, transfer of microbiota from treated mice reduced energy expenditure in untreated recipients, supporting a microbiota-dependent component of the metabolic phenotype [31]. More recent mechanistic work has extended these observations beyond energy metabolism. Early risperidone exposure in mice induced microbial dysbiosis, impaired intestinal barrier integrity, increased systemic LPS, and altered bile-acid and tyrosine metabolism; fecal microbiota transfer from risperidone-treated donors reproduced cognitive and synaptic abnormalities in recipient mice [32]. These findings provide direct experimental evidence that treatment-associated microbial communities can transmit components of an antipsychotic-related host phenotype, although their relevance to adult human treatment remains to be established. Human evidence remains predominantly associative. First exposure to second-generation antipsychotics has been associated with simultaneous alterations in gut microbial composition, fecal metabolites, glucose and lipid parameters, and body mass index [55]. During 24 weeks of risperidone treatment in drug-naive patients with first-episode schizophrenia, changes in *Bifidobacterium* abundance correlated with changes in body weight and body mass index [33]. Multi-omic studies have similarly linked antipsychotic-associated microbial features to weight gain and dyslipidemia [16]. In patients receiving long-term clozapine monotherapy, *Oscillibacter* abundance was associated with metabolic phenotype and correlated with triglyceride concentrations [37]. Proposed downstream involvement of PYY or GLP-1 remains mechanistically plausible but has not been established as a causal pathway in these patients. A vagal contribution has been demonstrated most directly in an olanzapine animal model. Olanzapine-induced microbial changes were accompanied by altered SCFAs, reduced intestinal serotonin, and increased hypothalamic NPY/AgRP signaling, whereas vagotomy attenuated weight gain and adipose accumulation [20]. Hypertriglyceridemia persisted despite vagotomy, indicating that vagal signaling accounts for only part of the metabolic phenotype. Disease-focused fecal-transfer studies provide complementary evidence that intestinal microbial communities can alter neuroactive pathways relevant to schizophrenia. Microbiota transferred from patients with schizophrenia altered glutamate-glutamine-GABA cycling and schizophrenia-relevant behaviors in recipient mice [14], whereas microbiota from drug-free patients produced behavioral abnormalities together with altered kynurenine metabolism and changes in neuroactive metabolites [15]. These experiments support the biological plausibility of microbiota-brain communication but do not establish that the same pathways mediate therapeutic or adverse effects of individual antipsychotics. Functional changes may provide greater mechanistic information than isolated taxonomic shifts. Longitudinal and multi-omic studies have linked antipsychotic-associated microbiome alterations to pathways involving carbohydrate and lipid metabolism, SCFAs, amino-acid metabolism, and other microbial metabolites [18,55]. For example, olanzapine-associated changes in SCFA-related pathways can be interpreted alongside weight gain, lipid metabolism, and inflammatory phenotypes rather than solely as changes in SCFA-producing taxa [18]. In risperidone models, altered secondary bile-acid and tyrosine metabolism, reduced TUDCA, increased p-cresol, impaired intestinal barrier integrity, and increased systemic LPS provide functional links between microbial remodeling and host effects [32]. Disease-focused microbiota-transfer studies also implicate tryptophan–kynurenine metabolism, although these findings are not drug-specific. Such findings support a shift from taxon-centered interpretations toward microbial functional networks and metabolite-mediated host responses, although comparable mechanistic data for most other antipsychotics remain sparse. The relationship between antipsychotic exposure and the microbiota is bidirectional. In rats, depletion of the gut microbiota increased systemic olanzapine exposure by approximately 80%, whereas risperidone exposure was not significantly altered [9]. This effect was accompanied by changes in intestinal glucuronidation-related pathways, and *Alistipes* abundance correlated with olanzapine exposure. These findings provide preclinical evidence for microbiota-dependent modulation of antipsychotic pharmacokinetics, but equivalent causal evidence in humans is currently lacking. Microbial profiles have also been associated with therapeutic response. In first-episode drug-naive schizophrenia, 24 weeks of risperidone monotherapy altered gut microbial composition, and several microbial features, including *Lachnoclostridium* and *Romboutsia*, were associated with subsequent symptom improvement [34]. In bipolar depression, metagenomic analysis identified microbial patterns that differentiated quetiapine responders from nonresponders within the study cohort [41]. Conversely, a shotgun-metagenomic study of treatment-resistant schizophrenia found no differentially abundant bacterial species between clozapine-responsive and clozapine-nonresponsive patients after adjustment for relevant covariates [38].

These response-associated microbial patterns require prospective external validation. The gut virome also represents emerging dimensions of antipsychotic–microbiome interactions. Metagenomic analysis identified extensive differences in gut viral communities between patients with schizophrenia and healthy controls; following three months of antipsychotic treatment, selected viral taxa changed, whereas broader virome differences were not fully normalized [56]. These observations suggest that viral components of the intestinal ecosystem may respond to treatment differently from bacterial communities, although molecule-specific effects remain unclear. Antipsychotic exposure may also exert selective pressure on antimicrobial resistance. Repeated quetiapine exposure promoted multidrug-resistant phenotypes in experimental *E. coli* populations and was associated with genomic and transcriptional changes affecting antibiotic susceptibility [42]. The clinical relevance of this mechanistic signal remains uncertain, but it raises potential implications for antimicrobial selection pressure and infection risk that require further investigation.

Sex, baseline microbial composition, disease stage, diet, and treatment history are major potential sources of heterogeneity. In human cohorts, atypical antipsychotic treatment was associated with reduced microbial diversity in women but not consistently in men [36,49], while clozapine-associated relationships between individual taxa and metabolic variables also differed according to sex [37]. These observations support consideration of sex as an effect modifier rather than assuming a uniform treatment-associated microbiome response. Disease stage and baseline microbial state further complicate causal attribution. First-episode drug-naive and chronically medicated patients with schizophrenia exhibit partly distinct microbial profiles [51], while studies in younger patients with schizophrenia-spectrum disorders demonstrate substantial disease-associated microbial differences even before treatment effects can be cleanly isolated [57]. In a prospective olanzapine-treated cohort, baseline community structure and metabolic potential varied between individuals despite relatively modest average taxonomic changes during follow-up [21].

Diet is another major modifier. Resistant-starch supplementation produced marked enrichment of selected *Bifidobacterium* taxa in patients receiving atypical antipsychotics, demonstrating that dietary substrate can substantially reshape the microbial community during pharmacological treatment [49]. Smoking, physical activity, constipation, hospitalization, polypharmacy, geography, and differences in microbiome methodology (including 16S rRNA sequencing, shotgun metagenomics, qPCR, and metabolomics) provide additional sources of between-study variability. Microbiota-directed interventions provide preliminary proof of concept but remain insufficiently validated for routine clinical use. In rats, the prebiotic Bimuno galacto-oligosaccharide (B-GOS) modified the intestinal microbiota and attenuated olanzapine-associated weight gain [22]. In humans receiving atypical antipsychotics, resistant-starch supplementation produced a pronounced bifidogenic response, although evidence for sustained metabolic benefit remains limited [49]. Clinical probiotic findings are heterogeneous. In patients beginning olanzapine treatment, Bifidobacterium supplementation reduced early weight gain at four weeks, but the between-group difference was no longer significant at eight or twelve weeks, suggesting that the effect may be transient [23]. In drug-naive patients with first-episode schizophrenia, probiotics alone did not significantly reduce olanzapine-associated weight gain, whereas the combination of probiotics and dietary fiber attenuated weight gain and insulin resistance in a separate randomized study within the same report [24]. A further double-blind randomized trial in patients with schizophrenia or bipolar disorder who had already gained more than 10% of body weight during atypical antipsychotic treatment found that probiotics combined with dietary fiber reduced weight, body mass index, and total cholesterol more effectively than either intervention alone or placebo [25]. Overall, the intervention literature remains limited by small sample sizes, short and variable follow-up periods, heterogeneity in probiotic strain selection and dietary formulations, differences in antipsychotic exposure, and inconsistent control of baseline diet and dietary changes. These factors may partly explain the divergent microbiome and metabolic outcomes reported across trials and limit direct comparison between studies. Microbiota-targeted approaches should therefore be regarded as experimental adjunctive strategies, and current evidence does not support their routine clinical use. Future trials should use drug-specific designs, longitudinal microbiome and metabolomic profiling, standardized cardiometabolic phenotyping, and appropriate control of dietary confounders.

## 3. Discussion

Overall, the available evidence suggests that antipsychotic-associated microbiome remodeling varies across individual compounds, with substantial differences in the strength and reproducibility of reported effects. Olanzapine has the most extensive mechanistic evidence, with germ-free, antibiotic-depletion, longitudinal, and vagotomy models linking microbiota alterations to weight gain, adiposity, inflammatory signaling, altered microbial metabolites, and lipid dysregulation [17,18,19,20]. Risperidone also induces reproducible treatment-associated microbial changes, but the direction of diversity and individual taxonomic shifts varies among populations, treatment durations, and experimental settings [31,33,34]. Clozapine-related findings are more difficult to disentangle from treatment resistance, gastrointestinal hypomotility, long illness duration, and metabolic syndrome [2,37,38]. Quetiapine has been associated with treatment-related taxonomic changes and candidate response-associated microbial patterns [36,41], whereas aripiprazole can influence intestinal microorganisms and gastrointestinal physiology without reproducing the characteristic metabolic burden associated with olanzapine [7,45]. Evidence for less extensively studied compounds remains insufficient for firm clinical conclusions. In a direct 21-day comparison in rats, lurasidone and olanzapine produced contrasting microbial and metabolic profiles: lurasidone was associated with increased microbial richness and alpha diversity and substantially less deterioration in weight, glucose, triglycerides, and inflammatory parameters [26]. Although these results raise the possibility of a comparatively microbiota- and metabolism-sparing profile, they remain preclinical and require independent replication in humans. This hierarchy of evidence is consistent with recent systematic syntheses showing considerable heterogeneity according to drug, study population, experimental model, sequencing method, and metabolic phenotype [27]. Population-specific studies further indicate that treatment-associated microbial patterns may not generalize across geographical settings; an Eastern European cohort, for example, identified microbial differences in antipsychotic-treated patients that overlapped only partially with patterns reported elsewhere [52]. Earlier systematic evidence similarly suggested that second-generation antipsychotics differ substantially in their microbiota-associated metabolic effects [28], while mechanistic reviews emphasize that the microbiome is more plausibly an interacting component of drug-induced metabolic dysfunction than a single common pathway shared by all compounds [29,30]. Accordingly, the term “antipsychotic dysbiosis” should be used cautiously. Neither an increased Firmicutes/Bacteroidota ratio, reduced alpha diversity, nor depletion of *Akkermansia* constitutes a universal treatment pattern. Even taxa conventionally considered beneficial may display context-dependent associations. *Bifidobacterium*, for example, increased during risperidone treatment in some cohorts, and its changes correlated with body weight and BMI [33]. Taxonomic direction alone therefore provides limited mechanistic information unless interpreted in relation to the specific drug, microbial community, host phenotype, and functional output. The strongest evidence that the intestinal microbiota contributes functionally to antipsychotic-associated phenotypes derives from experimental manipulation rather than observational taxonomic associations. Germ-free and colonization experiments indicate that the presence and composition of an intestinal microbial community contribute to olanzapine-associated weight gain [17]. Antibiotic depletion attenuates several olanzapine-induced metabolic abnormalities [19], whereas vagotomy separates vagus-dependent adiposity from metabolic effects that persist independently of this pathway [20]. With risperidone, transfer of treatment-associated microbiota can reduce energy expenditure in untreated recipient animals [31], and more recent fecal-transfer experiments reproduce intestinal, metabolic, and neurobiological components of risperidone exposure [32]. These models support a contributory role of the microbiota in selected host phenotypes, but not microbial exclusivity. Antipsychotics simultaneously act on central neurotransmitter receptors, peripheral metabolic tissues, gastrointestinal motility, appetite, endocrine pathways, and immune signaling. The microbiota should therefore be conceptualized as one component of an interacting drug-host-microbiome system, rather than as an alternative explanation replacing established pharmacological mechanisms. Human evidence remains substantially less causal. Longitudinal studies in antipsychotic-naive patients improve temporal inference because the pretreatment microbiome can be characterized before drug exposure [33,34], but treatment initiation often coincides with changes in diet, appetite, physical activity, sleep, bowel function, smoking, hospitalization, symptom severity, and concomitant medication use. Cross-sectional studies in chronically treated populations are even more susceptible to cumulative confounding and reverse causality. Meta-analytic evidence nevertheless supports measurable compositional and functional differences in schizophrenia and psychosis while simultaneously demonstrating marked between-study heterogeneity [10,12,58]. Broader meta-analysis across psychiatric disorders reaches a similar conclusion: microbiome alterations are detectable at the group level, but reproducibility of individual taxa is limited by diagnostic, geographical, lifestyle, and methodological variation [59]. A major implication of this synthesis is that future studies should move beyond the search for individual “beneficial” or “harmful” bacterial taxa. Microbial metabolites and functional pathways (including SCFAs, bile acids, tryptophan-kynurenine metabolites, aromatic amino-acid products, LPS-associated signaling, and microbial drug-transforming enzymes) are more directly connected to host physiology than relative abundance alone [9,16,32]. Shotgun metagenomics in schizophrenia has identified abnormalities in SCFA-producing bacteria together with predicted functional disturbances in glucose and lipid metabolism [60]. Longitudinal and multi-omic antipsychotic studies similarly indicate that microbial metabolic trajectories may track weight gain and other metabolic outcomes more closely than individual taxonomic changes [16,18]. These observations argue for an analytical shift from “which bacterium changes?” to “which microbial function changes, by how much, and with what host consequence?” An additional methodological limitation is that most microbiome studies report relative abundance. Relative changes cannot distinguish true expansion of one microbial population from depletion of surrounding community members. Quantitative microbiome profiling has demonstrated that variation in total microbial load can substantially modify the interpretation of taxonomic associations and ecological relationships [61]. Future studies should therefore incorporate absolute microbial quantification when feasible and clearly differentiate predicted functional capacity from measured microbial genes, transcriptional activity, and directly quantified metabolites. Anatomical resolution is equally important. Fecal samples predominantly represent distal luminal communities and may not adequately capture the small intestine or mucosal compartment, where drug absorption, bile-acid exposure, serotonin signaling, and epithelial permeability may differ. The region-specific increase in ileal permeability observed after psychotropic exposure illustrates how biologically relevant effects can be missed by stool analysis alone [7]. Repeated early sampling is also necessary because microbial changes may emerge within days of treatment and subsequently fluctuate, partially normalize, or be obscured by downstream metabolic changes [18,33]. For a microbiome-based measure to have clinical value, it should predict a predefined and actionable outcome (such as substantial weight gain, insulin resistance, constipation, or therapeutic response) before the outcome occurs and provide information beyond readily available clinical predictors. Several studies provide proof of concept. Baseline or treatment-associated microbial profiles have been associated with risperidone response [34], quetiapine response [41], and antipsychotic-induced weight gain in longitudinal multi-omic datasets [16]. However, most candidate classifiers remain internally derived from relatively small cohorts and have not undergone prospective external validation. Population characteristics are likely to influence predictive performance. Preliminary pediatric evidence suggests associations between atypical-antipsychotic-associated overweight and specific microbial profiles in Asian children [62]. Independent work in schizophrenia has explored microbial patterns as potential non-invasive classifiers distinguishing patients from controls [63], while other studies have associated gut microbial alterations with symptom severity [64]. Recent systematic synthesis of biomarker studies likewise concludes that taxonomic and beta-diversity signals are promising but insufficiently reproducible for clinical deployment [65]. The relevant clinical phenotype may also extend beyond metabolic outcomes. Microbiome–gut–brain profiles have been associated with cognitive performance in schizophrenia [66], and short-term antipsychotic treatment has been linked to changes in microbial co-occurrence networks and specific taxa [35]. Such observations raise the possibility that microbiome-informed stratification could eventually encompass cognition or treatment response in addition to cardiometabolic toxicity, although these applications remain exploratory. At present, no microbiome-derived biomarker, response classifier, or microbiota-targeted intervention has been prospectively validated for routine clinical use in antipsychotic-treated patients. Cross-diagnostic evidence further cautions against assuming that all treatment-associated effects are specific to schizophrenia. A systematic review of psychotropic treatment in bipolar disorder identified microbiome changes across several pharmacological exposures [43]. Gut microbiota remodeling has also been reported in adolescents with bipolar depression in association with sensory-emotional functional abnormalities [67], while earlier metagenomic work identified distinct microbial alterations in adults with bipolar depression [44]. These studies support investigation of drug-related microbial effects across diagnostic boundaries, but they also introduce additional heterogeneity related to mood state, age, concomitant treatment, and underlying disease biology. Treatment resistance represents another clinically important context. Reviews and primary studies indicate that microbiome alterations in treatment-resistant schizophrenia may reflect a mixture of disease severity, long-term medication exposure, clozapine treatment, constipation, diet, and metabolic burden [38,39]. Consequently, microbial differences between treatment-responsive and treatment-resistant populations cannot be attributed to treatment response alone. Gastrointestinal effects may also have consequences beyond metabolic dysfunction. Clozapine-associated gastrointestinal hypomotility, including severe constipation and potential ileus, requires careful monitoring of bowel function. Furthermore, recent experimental work with clozapine suggests that gastrointestinal hypomotility and microbiota-metabolite alterations may interact with extraintestinal systems, including the gut-lung axis [40]. Although such findings remain preclinical, they broaden the clinical perspective beyond weight gain and glucose-lipid metabolism and reinforce the importance of monitoring gastrointestinal physiology as an integral component of antipsychotic safety. A rigorous precision pharmacomicrobiomic study would therefore enroll antipsychotic-naive patients initiating a single defined compound, obtain baseline stool and blood before treatment, and repeat sampling during the first days, weeks, and months. Diet, physical activity, smoking, bowel function, hospitalization, and concomitant medications should be repeatedly measured rather than treated as static covariates. Microbial sequencing should ideally be combined with targeted SCFAs, bile acids, tryptophan metabolites, inflammatory and endocrine markers, standardized cardiometabolic phenotyping, and antipsychotic plasma concentrations. Drug formulation should also be considered. Experimental pharmaceutical studies with lurasidone show that formulation strategies capable of modifying oral bioavailability can simultaneously alter the intestinal microbial environment [46,47]. Thus, luminal drug exposure is determined not only by the active molecule and dose but also by formulation, absorption, and gastrointestinal disposition. Future comparison of oral and long-acting injectable preparations may therefore help distinguish direct luminal drug-microbe interactions from systemic host-mediated effects. Microbiota-directed approaches should currently be regarded as adjunctive experimental strategies rather than alternatives to effective antipsychotic therapy and should not be used to modify antipsychotic exposure or metabolic outcomes without appropriate clinical monitoring. Reviews of microbiota-targeted approaches in schizophrenia have proposed probiotics, prebiotics, synbiotics, dietary modulation, and metabolite-directed strategies, but emphasize the limited clinical evidence and considerable heterogeneity of available studies [48,68]. Non-pharmacological approaches intended to modify microbial diversity have likewise been proposed, but their effects remain highly dependent on intervention type, host diet, baseline microbiome, and treatment context [69]. Experimental and clinical studies nevertheless provide preliminary proof of concept. In rats, Bimuno galacto-oligosaccharide modified the intestinal microbiota and attenuated olanzapine-associated weight gain [22]. In patients initiating olanzapine, *Bifidobacterium* supplementation transiently attenuated early weight gain, although the between-group effect was not sustained throughout follow-up [23]. Randomized studies combining probiotics with dietary fiber reported more favorable metabolic outcomes than probiotics alone in selected antipsychotic-treated populations [24,25]. These findings require confirmation in larger and longer-term studies. The overall clinical literature remains mixed. A recent systematic review and meta-analysis concluded that available evidence is insufficient to recommend microbiota manipulation routinely as a treatment strategy in schizophrenia [70]. A subsequent meta-analysis of randomized controlled trials reported signals of improvement in psychopathology and selected glucose- and lipid-related outcomes but did not demonstrate consistent reductions in body weight or BMI [71]. No standardized probiotic, prebiotic, synbiotic, postbiotic, or dietary strategy is currently established for antipsychotic-associated metabolic dysfunction. Current evidence does not support a universal probiotic strategy across antipsychotic-treated populations. Individual antipsychotics differ in luminal exposure, metabolic liability, gastrointestinal effects, and microbial associations, while baseline microbial communities vary substantially among individuals and populations. Future interventions should therefore be drug-specific, function-guided, and linked to clearly defined outcomes (for example, prevention of early weight gain, preservation of SCFA production, treatment of constipation, restoration of epithelial barrier function, or attenuation of inflammatory signaling). Postbiotics and defined microbial metabolites may eventually provide greater compositional standardization than live organisms, but their efficacy, optimal dosing, and safety in antipsychotic-treated populations remain to be demonstrated. Equally important, microbiota manipulation could alter drug disposition. In rats, depletion of the gut microbiota increased systemic olanzapine exposure despite attenuating several metabolic effects [9]. Therefore, an intervention that improves microbial or metabolic parameters could theoretically modify antipsychotic exposure in an unintended direction. Future trials should incorporate therapeutic drug monitoring, pharmacokinetic endpoints, and systematic adverse-effect surveillance whenever a plausible microbiota-drug interaction exists.

### Limitations

Several limitations should be considered when interpreting the present synthesis. First, the evidence base is highly heterogeneous with respect to psychiatric diagnosis, antipsychotic compound, dose, treatment duration, previous medication exposure, age, sex, metabolic phenotype, diet, geographical setting, biological sampling time, and microbiome methodology. Direct comparisons between individual drugs are therefore frequently indirect.

Second, human evidence remains dominated by relatively small observational studies, whereas the strongest causal evidence derives from animal, germ-free, antibiotic-depletion, vagotomy, and fecal-transfer models. These experimental approaches provide mechanistic resolution that cannot be achieved in observational human studies but cannot be assumed to translate quantitatively to clinical populations.

Third, microbiome methodology remains heterogeneous. Many studies rely on 16S rRNA sequencing and relative taxonomic abundance, and comparatively few integrate shotgun metagenomics, absolute microbial quantification, metabolomics, host biomarkers, antipsychotic concentrations, and repeated metabolic phenotyping. Consequently, functional interpretations are often inferred rather than directly measured.

Fourth, disease- and treatment-associated effects remain difficult to separate completely. Schizophrenia itself, disease stage, hospitalization, diet, smoking, gastrointestinal symptoms, metabolic phenotype, and psychotropic polypharmacy may influence the microbiome independently of the current antipsychotic. This limitation is particularly important in chronically treated and treatment-resistant populations.

Finally, because this narrative review synthesizes a heterogeneous evidence base, the coverage and comparative weighting of studies may have been influenced by the selection of sources.

## 4. Methods

### 4.1. Review Design

This narrative review integrates evidence from human observational and interventional studies, animal and gnotobiotic models, fecal microbiota transplantation experiments, in vitro investigations, pharmacokinetic studies, and multi-omic analyses. A narrative approach was selected because the available literature is highly heterogeneous with respect to diagnosis, antipsychotic exposure, treatment duration, sampling strategy, microbiome methodology, and reported outcomes.

### 4.2. Information Sources and Search Strategy

A literature search was conducted in PubMed/MEDLINE, Web of Science, and Scopus, primarily covering studies published from January 2016 to July 2026. Earlier seminal studies were additionally included when they provided unique mechanistic or causal evidence that had not been superseded by more recent investigations. Search strategies combined microbiome-related terms (“gut microbiome”, “gut microbiota”, “intestinal microbiome”, “intestinal microbiota”, and “gut-brain axis”) with antipsychotic-related terms (“second-generation antipsychotics”, “atypical antipsychotics”, “third-generation antipsychotics”, “antipsychotics”, and “neuroleptics”). Additional searches included terms related to metabolic adverse effects (e.g., “weight gain”, “metabolic syndrome”, “insulin resistance”, “dyslipidemia”), intestinal permeability (e.g., “intestinal barrier”, “gut permeability”), inflammation (e.g., “inflammation”, “lipopolysaccharide”, “LPS”), microbial metabolites (e.g., “short-chain fatty acids”, “SCFAs”, “bile acids”, “tryptophan metabolites”), gastrointestinal motility (e.g., “constipation”, “gastrointestinal hypomotility”, “intestinal transit”), and microbiota-targeted interventions (e.g., “probiotics”, “prebiotics”)

### 4.3. Literature Selection for the Narrative Synthesis

Studies were included if they:

(i) investigated one or more antipsychotic drugs or a clearly defined antipsychotic treatment category;

(ii) evaluated the intestinal microbiota or a directly related microbial functional outcome;

(iii) reported outcomes related to microbial composition or diversity, microbial metabolites, intestinal barrier function, inflammation, metabolic adverse effects, pharmacokinetics, treatment response, or microbiota-targeted interventions;

(iv) used human, animal, gnotobiotic, fecal microbiota transplantation, or relevant in vitro models;

(v) were published in English as full-length, peer-reviewed articles.

Studies were excluded if they focused exclusively on non-intestinal microbial communities, did not provide information relevant to antipsychotic exposure or the objectives of the review, or were editorials, letters, commentaries, dissertations, book chapters, or other non-peer-reviewed publications. Conference abstracts, trial protocols, and preliminary reports without sufficient outcome data were excluded from the core evidence synthesis. Records were screened by title and abstract, followed by full-text assessment of potentially eligible publications. Duplicate records were removed before synthesis. The combined database searches yielded 100 records. After removal of 12 duplicates, 88 records were reviewed based on title and abstract, and 5 were excluded at this stage. Full-text retrieval was sought for 83 publications, of which 3 could not be retrieved. Eighty publications were assessed in full text, and 9 were excluded because they addressed conditions outside the scope of the review. The remaining 71 publications contributed to the final narrative synthesis.

### 4.4. Interpretation of Evidence

Evidence was interpreted according to study design. Human longitudinal studies were used to assess changes over time, while cross-sectional studies were used to describe associations at a single time point. Animal, germ-free, colonization, antibiotic-depletion, vagotomy, and fecal microbiota-transfer studies were used to explore mechanisms. In vitro studies were used to assess direct drug–microbe effects. Multi-omic studies were used to link microbial changes with functional pathways and metabolites. No formal risk-of-bias assessment was performed.

During the preparation of this manuscript, the authors used OpenAI to assist with language refinement, clarity, restructuring, condensation of selected passages, and generating the conceptual illustration presented as Figure 1. The tool was not used for literature selection, data generation, data analysis, or independent interpretation of the scientific evidence. All AI-assisted text was critically reviewed and revised by the authors, who take full responsibility for the content of the manuscript.

## 5. Conclusions and Future Perspectives

Current evidence supports a bidirectional relationship between antipsychotic treatment and the gut microbiota but does not indicate a uniform pattern of microbiome alteration across compounds. Drug-specific effects appear to differ in magnitude, direction, and functional relevance, with olanzapine currently supported by the strongest mechanistic evidence and risperidone also showing microbiota-dependent metabolic and neurobiological effects in preclinical models. For most other antipsychotics, the evidence remains heterogeneous or limited. Overall, microbial function may be more informative than isolated taxonomic changes. Integrating microbial composition with functional outputs such as SCFAs, bile acids, tryptophan-derived metabolites, barrier and inflammatory pathways, and microbiota-dependent drug metabolism may provide greater mechanistic and translational insight. Future studies should prioritize prospective, longitudinal, drug-specific designs in antipsychotic-naive patients, with repeated microbiome sampling, multi-omic profiling, cardiometabolic phenotyping, and therapeutic drug monitoring. Microbiota-based biomarkers and interventions remain promising but experimental and require external validation and randomized clinical evaluation before they can be incorporated into precision antipsychotic treatment.

## Figures and Tables

**Figure 1:**
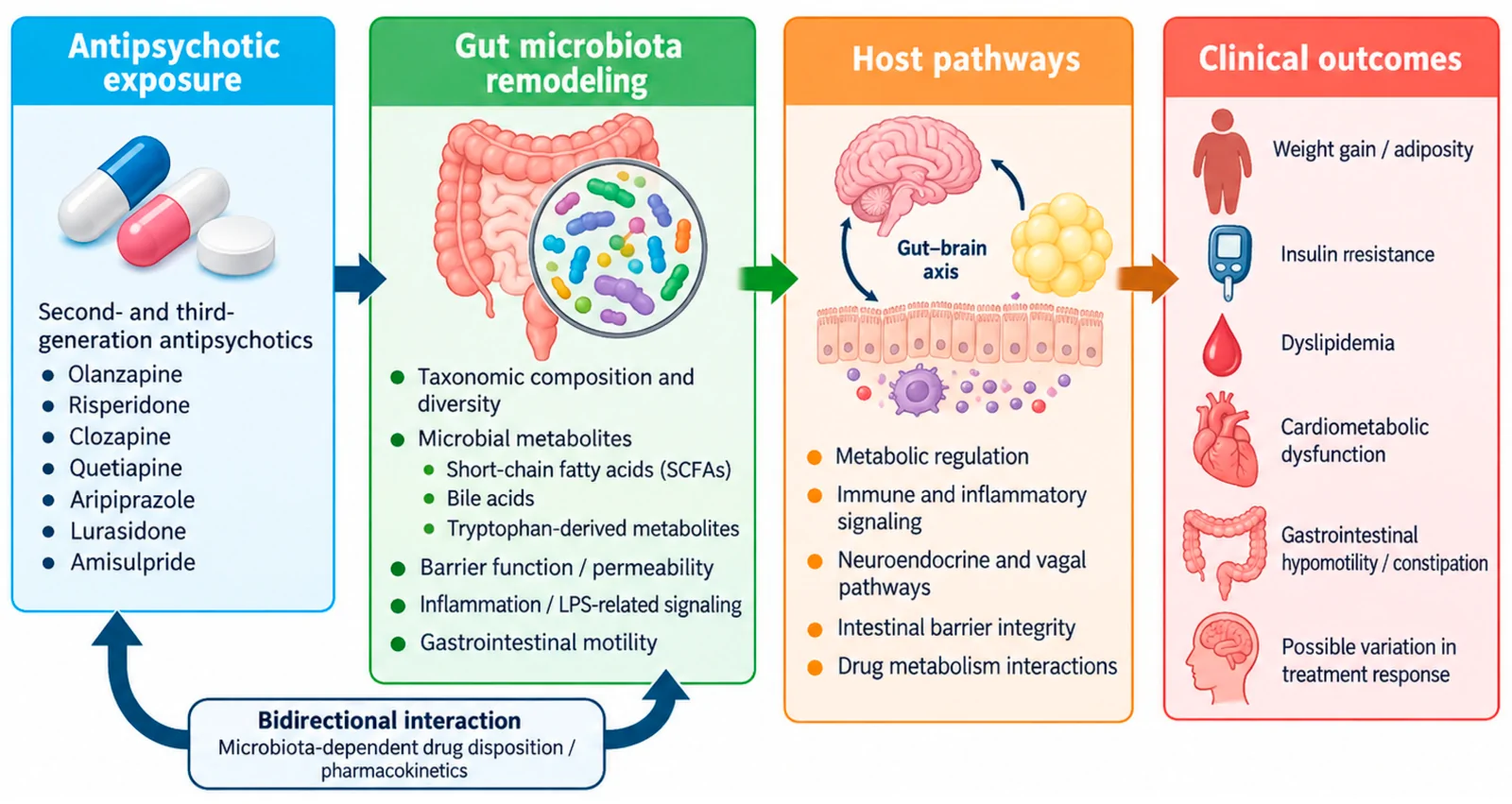
**Conceptual framework of antipsychotic–gut microbiota interactions.** Antipsychotic exposure may contribute to gut microbiota remodeling at the levels of taxonomic composition, microbial metabolites, barrier function, inflammation, and gastrointestinal motility. These changes may influence host pathways involved in metabolic regulation, immune and inflammatory signaling, neuroendocrine and vagal communication, intestinal barrier integrity, and drug metabolism, with potential consequences for weight gain, insulin resistance, dyslipidemia, cardiometabolic dysfunction, gastrointestinal hypomotility, and treatment response. The framework also illustrates the bidirectional relationship whereby the gut microbiota may influence antipsychotic disposition and pharmacokinetics. The figure summarizes the conceptual model discussed in this review and does not imply uniform effects across all antipsychotic compounds. The figure was generated with the assistance of OpenAI (ChatGPT-5.6).

**Table 1:** Drug-specific gut microbiota patterns reported for antipsychotics in the reviewed corpus.

Agent	Reported Microbial Pattern	Related Host Effects	Notes	Source Key
**Olanzapine**	↑ Firmicutes/Bacteroidota ratio (animal) ↑ *Erysipelotrichia*/Erysipelotrichaceae ↑ Gammaproteobacteria; ↓ Bacteroidia/Bacteroidota, ↓ *Akkermansia muciniphila*, ↓ *Blautia*, ↓ Lachnospiraceae, ↓ *Lachnoclostridium* ↓ *Parasutterella*. ↓ *E. coli* (completely inhibited at high concentration in vitro). ↓ Actinobacteria (especially women) ↓ Proteobacteria (especially women) ↑ *Erysipelotrichia* ↑ Gammaproteobacteria In rodent models, olanzapine frequently produces reduced microbial richness/alpha diversity and total OTUs, compositional shifts involving *Firmicutes* and *Bacteroidota*, and, in several studies, an increased Firmicutes/Bacteroidota ratio. Depletion of *Akkermansia muciniphila* and selected SCFA-associated taxa has been reported, although taxonomic direction is not uniform across models.	Weight gain and adiposity; glucose and lipid dysregulation; inflammatory and hepatic lipogenic signaling; altered intestinal 5-HT and hypothalamic NPY/AgRP pathways. Germ-free/colonization, antibiotic-depletion and vagotomy experiments support a contributory role of the microbiota in part of the metabolic phenotype. Microbiota-targeted adjuncts have attenuated early weight gain in selected experimental and clinical studies.	Strongest mechanistic evidence among antipsychotics, but no universal microbiome pattern The Firmicutes/Bacteroidota ratio and SCFA direction are model-dependent. Human evidence remains more heterogeneous than preclinical evidence. Strong preclinical support from germ-free/colonization, antibiotic-depletion, and vagotomy models. Human longitudinal evidence is available but remains heterogeneous. Main confounders include diet, sex, baseline microbiota, and treatment duration. A reproducible, drug-specific human microbiome pattern remains unproven.	[3,7,9,16,17,18,19,20,21,22,23,24,25,26,27,28,29,30]
**Risperidone**	↑ Erysipelotrichaceae (animal) ↑ Mollicutes (animal) ↓ *Alistipes* ↓ *Akkermansia* ↑ Lachnospiraceae ↓ *Subdoligranulum* ↑ *Clostridium sensu stricto-1* ↑ *Clostridium UCG005* ↑/↓ *Bifidobacterium* spp., ↓ *Lactobacillus* spp., ↑ *Escherichia coli*, ↓ *Clostridium coccoides*, ↓ *Lachnoclostridium*, ↑ *Romboutsia*, ↑ *Ruminococcus* ↑ *Eubacterium siraeum*. ↑ *Allobaculum* ↑/↓ *Firmicutes*/*Bacteroidetes* ratio Direction differs markedly across populations and durations.	Weight/BMI gain; glucose, triglyceride, LDL, HOMA-IR and inflammatory changes; reduced energy expenditure. Fecal microbiota transfer (FMT) from risperidone-treated animals reduced resting energy expenditure in untreated recipients. Recent experimental work also links risperidone-associated microbiota to barrier dysfunction and altered bile-acid/aromatic-amino-acid metabolism.	Relatively strong longitudinal and mechanistic evidence, but taxonomic results are inconsistent. *Bifidobacterium* is a clear example of a taxon whose increase cannot automatically be interpreted as beneficial. In first-episode drug-naive patients, 24-week treatment produced ↑ *Bifidobacterium* spp. and *E. coli* and ↓ *Lactobacillus* spp. and *Clostridium coccoides*. Dedicated paliperidone evidence remains insufficient. Preclinical fecal-transfer studies support a contributory role of the microbiota. Human longitudinal evidence is available, including drug-naive first-episode cohorts, but taxonomic findings remain inconsistent. Disease stage, baseline microbiota, and treatment duration are important confounders. A clinically validated microbiome predictor remains unproven.	[3,10,27,28,30,31,32,33,34,35]
**Clozapine**	↑ Proteobacteria ↑ *Firmicutes* ↑ *Bacteroidetes* ↑ Actinobacteria ↓ *Oscillibacter* Sex-dependent associations involving *Gemmiger* and *Bifidobacterium*. Reviews report ↑ Lachnospiraceae ↓ *Akkermansia* ↓ *Sutterella*. Long-term clozapine monotherapy is associated with differences in microbial diversity and composition according to sex and metabolic phenotype.	Associations with triglycerides and glycemic markers; strong interaction with metabolic syndrome, treatment resistance, and gastrointestinal hypomotility. *Oscillibacter* is reduced/low in abundance and shows reproducible associations with glucose-lipid variables; *Gemmiger* and *Bifidobacterium* display sex-dependent metabolic associations. A 2026 mouse study linked clozapine-induced gastrointestinal hypomotility to region- and sex-dependent microbiota/metabolite alterations and increased vulnerability to experimental lung injury.	Interpretation is strongly confounded by treatment resistance, long illness duration, constipation, polypharmacy, and metabolic syndrome. Current data do not support a reproducible microbiome pattern separating clozapine responders from nonresponders. Broad changes in Lachnospiraceae, *Akkermansia*, and *Sutterella* have been described in mixed atypical-antipsychotic cohorts but should not be attributed specifically to clozapine. Mechanistic evidence is limited and remains largely preclinical. Human evidence is mainly observational. Interpretation is strongly confounded by treatment resistance, long illness duration, constipation, polypharmacy, and metabolic syndrome. A reproducible microbiome pattern associated with treatment response remains unproven.	[2,27,30,36,37,38,39,40]
**Quetiapine**	↑ Proteobacteria, ↑ *Klebsiella*, ↑ *Lactobacillus*, ↑ *Anaeroglobus,* ↑ *Collinsella,* ↑ *Paraprevotella,* ↑ *Solobacterium* ↑ *Veillonella* ↓ *Alistipes* ↑ *Streptococcus cristatus*, ↑ *Clostridium perfringens* ↑/↓ *C. bartlettii*. ↑ *E. coli* ↓ *Bilophila,* ↓ *Enterococcus hirae* ↓ *Bifidobacterium dentium* ↑ *Odoribacter splanchnicus*, ↑ *Oribacterium sinus*, ↑ *Hafnia alvei*, ↑ *Fusobacterium periodonticum*, ↑ *Acidaminococcus interstini* ↑ *Veillonella rogosae* Metagenomic studies identified additional species-level differences and candidate responder-associated patterns. Alpha-diversity findings vary across studies and treatment stages.	Candidate microbial patterns have been associated with treatment response, depressive severity and neuroimaging measures. Experimental work also suggests that repeated quetiapine exposure can promote antimicrobial-resistance phenotypes in *E. coli*.	Evidence is derived largely from small bipolar-depression cohorts, limiting extrapolation to schizophrenia. Predictive microbiome models require independent external validation. Causal microbiota-manipulation evidence is lacking. Human evidence is limited mainly to small bipolar-depression cohorts, with some metagenomic findings. Diagnosis, sample size, and treatment context are important confounders. Response-associated microbial patterns require external validation.	[27,36,41,42,43,44]
**Aripiprazole**	↑ Firmicutes/Bacteroidota ratio (animal) ↓ *Lactobacillus casei* ↓ *E. coli 4* ↓ *Enterococcus faecium4* ↓ *Bacteroides fragilis* ↓ *Clostridium leptum* ↓ *Ruminococcus 1* ↑ Peptostreptococcaceae ↑ *Clostridiaceae* ↑ Ruminococcaceae ↑ *Ruminiclostridium 5*, ↑ *Intestinibacter*, ↑ *Eubacterium coprostanoligens*, ↑ *Peptoclostridium* ↑ *Clostridium sensu stricto 1* In rats, aripiprazole produced increased microbial richness/diversity, with changes involving Peptostreptococcaceae, Clostridiaceae, Ruminococcaceae, and several related genera. In vitro, aripiprazole inhibited several commensals, including *A. muciniphila*, *Lactobacillus*/*Lacticaseibacillus casei*, *Enterococcus faecium*, *Bacteroides fragilis*, and *Clostridium leptum*, but at relatively high concentrations. Cecal acetate and isovalerate were altered.	Increased distal ileal permeability without corresponding colonic permeability changes was observed in rats. Changes in microbial composition and SCFAs occurred without the pronounced metabolic phenotype associated with olanzapine.	The primary Cussotto study reported increased species richness/diversity. Direct antimicrobial effects occur at concentrations that may exceed clinically relevant luminal exposure, so in vitro and in vivo effects should be distinguished. Evidence is mainly preclinical and in vitro, with no strong human longitudinal microbiome evidence. Dose, experimental model, and clinically relevant intestinal exposure are important limitations. The clinical relevance of the reported microbial changes remains unproven.	[6,7,27,43,45]
**Lurasidone**	↑ *Lachnoclostridium* ↑ *Faecalibacterium* In the principal 21-day comparative rat study, lurasidone produced ↑ OTUs/richness and ↑ Shannon alpha diversity, whereas olanzapine produced the opposite pattern. The Firmicutes/Bacteroidota ratio was not significantly altered, and total SCFA concentrations were preserved relative to controls and were higher than in olanzapine-treated animals.	No significant weight gain or major deterioration in glucose, triglycerides, or inflammatory measures was observed in that model.	Suggests a potentially microbiota- and metabolism-sparing (“gut-neutral”) profile, but this conclusion currently rests predominantly on preclinical evidence. Pharmaceutical formulation can also modify lurasidone bioavailability and microbiota effects, complicating attribution to the molecule alone. Evidence is predominantly preclinical, mainly from comparative animal studies. Human longitudinal microbiome evidence is lacking. Pharmaceutical formulation may influence both drug exposure and microbiota effects. A microbiota-sparing profile in humans remains unproven.	[26,46,47]
**Amisulpride**	↑ *Dorea*, ↑ *Butyricicoccus*, ↑ *Actinomyces* ↑ *Porphyromonas*, No significant change in overall community richness or alpha diversity was detected. Predicted butanoate metabolism decreased after treatment.	Increased IL-4 and decreased IL-6 were reported alongside the microbial changes, suggesting an association with immune and SCFA-related pathways; the biological significance remains uncertain.	Evidence is very sparse and based essentially on one small clinical study. The primary amisulpride study is summarized in systematic/review literature in the current bibliography rather than listed separately as a standalone reference. No strong causal microbiota-manipulation evidence is available. Human evidence is very limited and based on a small clinical study. Small sample size and limited replication restrict interpretation. Reproducibility and clinical relevance remain unproven.	[27,29,48]
**Other/mixed agents**	Mixed atypical-antipsychotic cohorts show community separation from untreated patients and, in some studies, lower diversity predominantly in women, with differences involving Lachnospiraceae, *Akkermansia*, and *Sutterella*. Studies comparing drug-naive and chronically medicated schizophrenia demonstrate overlapping disease- and treatment-associated patterns. In vitro screening indicates that non-antibiotic psychotropics, including several antipsychotics, can exert direct bacterial growth pressure.	Potential effects include metabolic dysfunction, gastrointestinal physiology, microbial drug metabolism, and antimicrobial-resistance selection. However, molecule-level attribution is usually impossible in mixed-regimen cohorts.	First-generation antipsychotics may exert stronger off-target antimicrobial effects than many SGAs, but evidence is assay-dependent. Dedicated microbiome evidence remains limited for ziprasidone and paliperidone and is insufficient for firm conclusions regarding cariprazine or brexpiprazole.	[6,10,27,28,29,30,36,43,49,50,51,52]

↑, increase; ↓, decrease; ↑/↓, mixed direction across studies. 5-HT, serotonin; BMI, body mass index; FMT, fecal microbiota transplantation; HOMA-IR, homeostatic model assessment of insulin resistance; NPY/AgRP, neuropeptide Y/agouti-related peptide; OTU, operational taxonomic unit; SCFAs, short-chain fatty acids. Arrows indicate the direction reported in the cited studies and should not be interpreted as universal drug effects. Taxonomic nomenclature was checked against currently accepted naming conventions. Genus and species names are italicized throughout the manuscript. Where older taxonomic names were used in the original studies, the currently accepted terminology is used when appropriate; for example, Bacteroidetes and Firmicutes correspond to Bacteroidota and Bacillota, respectively. The Firmicutes/Bacteroidota ratio is reported as a context-dependent ecological descriptor and should not be interpreted as a universal biomarker or as a standalone indicator of clinical risk.

## Data Availability

No new data were created or analyzed in this study. Data sharing is not applicable to this review article.
